# Healthcare establishments as owner-operators of digital billboards: making the most of excellent roadside visibility and high traffic counts to better connect with patients

**DOI:** 10.1186/s12913-018-3680-y

**Published:** 2018-12-14

**Authors:** James K. Elrod, John L. Fortenberry

**Affiliations:** 1 Willis-Knighton Health System, 2600 Greenwood Road, Shreveport, LA 71103 USA; 2 LSU Shreveport, 1 University Place, Shreveport, LA 71115 USA

**Keywords:** Billboard advertising, Digital billboards, Outdoor advertising, Marketing communications, Healthcare

## Abstract

**Background:**

Given the importance of communicating effectively with current and prospective patients, healthcare institutions must direct considerable energies toward achieving associated excellence. Pursuits usually center on addressing the marketing communications mix properly, but in order to maximize communications prowess, attention also should be directed toward incorporating emerging communications innovations, when and where possible, to bolster opportunities to connect with patients.

**Discussion:**

In pursuit of communications excellence, Willis-Knighton Health System’s executives reflected on campus dynamics in the context of modern outdoor advertising technologies, namely, digital billboards, which present electronic advertisements on-demand, around-the-clock. They surmised that, with Willis-Knighton Health System’s campuses being positioned in highly-visible fashion along highly-transited roadways, the institution could deploy this relatively new roadside advertising innovation to better engage audiences. This particular train of thought ultimately led to the installation of digital billboards at several of its locations. This article profiles the development of Willis-Knighton Health System’s new marketing communications asset and offers advice for healthcare establishments desirous of placing digital billboards onsite to better connect with patients.

**Conclusions:**

Opportunities affording enriched communications with patients should continually be sought by health and medical entities. Digital billboards, institutionally-owned and operated, supply one such opportunity, pairing a state-of-the-art, uniquely-capable advertising medium with the excellent locational characteristics possessed by many healthcare establishments. As Willis-Knighton Health System has observed, digital billboards offer an exceptional mechanism for engaging audiences, affording mutual benefits which ultimately foster patronage and prosperity.

## Background

Communicating successfully with current and prospective patients represents an essential and ongoing task for healthcare providers [1, 2]. Doing so is important for a variety of reasons, especially for purposes of advising and informing audiences of details that can prove beneficial to their health and well-being, such as medical service availabilities, health screening opportunities, healthy lifestyles guidance, and so on [3–6]. But successful communications benefit not only patients; they also benefit the health and medical institutions disseminating the given messages. Competition within the healthcare industry is intense, with each provider being desirous of capturing the patronage of what very often are limited pools of patients, magnifying rivalry between and among institutions [2, 7, 8]. Such competitive environments demand that healthcare institutions communicate effectively with their audiences, placing premiums on the ability to carefully craft and convey appeals that compel patients to direct patronage inward toward given institutions, rather than outward toward rivals. As such, communications excellence is essential for ensuring institutional viability and prosperity [9–11].

In pursuit of excellence in communications, attention usually is centered on addressing the marketing communications mix proficiently, ensuring that proper selections are made from its five components of advertising, personal selling, public relations, direct marketing, and sales promotion, engaging desired audiences well and fostering their associated patronage [2, 9]. Formulation of the marketing communications mix indeed is a vital pursuit, but in order to maximize communications prowess, attention also should be directed toward incorporating emerging communications innovations, when and where possible, to bolster opportunities to connect with patients. Willis-Knighton Health System happened upon one such innovation while reflecting on campus dynamics in the context of modern outdoor advertising technologies, namely, digital billboards.

Digital billboards are modern renditions of traditional billboards, those large, stationary structures situated along transit pathways which display advertisements to passersby [2, 12, 13]. Replacing static panels which feature printed advertisements with massive digital panels which feature electronic advertisements, digital billboards have greatly advanced the communications utility of this classic advertising medium. Of particular benefit, the electronic format of digital billboards permits advertisements to be changed on-demand, remotely via computer. Coupled with their vibrant, full-color displays, digital billboards significantly improve upon the capabilities of their static counterparts. Given that the number of miles traveled via roadway by consumers continues to increase [14], billboards are particularly well positioned to play an increasing role in delivering advertising content to the general public [15, 16], with their digital variants providing advancements which will only help to derive the most from this historic medium of communication.

Although they typically are owned and operated by outdoor advertising companies which, in turn, lease space to those wishing to deliver advertising messages at given locations [12, 17, 18], digital billboards featuring like specifications can be purchased and operated by most any entity possessing a viable location for placement and appropriate resources. Willis-Knighton Health System recognized the ideal locations of its campuses, each situated prominently on roadways with exceptionally high traffic counts, realizing that these were perfect environments for digital billboards. After gaining an extensive understanding of digital billboard use via leased arrangements with outdoor advertising companies, coupled with careful research and reflection on the prospect of institutional ownership and operation of these advertising platforms, Willis-Knighton Health System installed digital billboards at several of its locations, affording significant improvements in the institution’s ability to connect with patients. This article profiles the development of this new marketing communications asset and offers advice for healthcare entities desirous of placing digital billboards onsite to foster patient attention and engagement.

## Willis-Knighton Health System and its history and use of billboard advertising

Headquartered in Shreveport, Louisiana, Willis-Knighton Health System is a nongovernmental, not-for-profit healthcare provider delivering comprehensive health and wellness services through multiple hospitals, numerous general and specialty medical clinics, an all-inclusive retirement community, and more. The system holds market leadership in its served region, centered in the heart of an area known as the Ark-La-Tex, where the states of Arkansas, Louisiana, and Texas converge. Willis-Knighton Health System’s origins date to 1924 with the establishment of Tri-State Sanitarium, founded to address the healthcare needs of the burgeoning population of west Shreveport. Sold in 1929 to Drs. James Willis and Joseph Knighton, the establishment continued operations and, in 1952, it was renamed in honor of Drs. Willis and Knighton. Going forward, the institution concentrated exclusively on serving the population of west Shreveport, but in the 1970s, expansion initiatives beyond this particular locale were pursued. These initiatives led to dramatic growth in ensuing decades, eventually resulting in comprehensive market coverage and market leadership [19, 20].

The fantastic growth experienced by Willis-Knighton Health System over the past several decades can be credited in part to the institution’s efforts to successfully engage current and prospective patients in the marketplace, with the consistent development and deployment of effective marketing communications anchoring associated efforts. Known for its ability to identify innovations emerging outside of the healthcare industry for use within [21], Willis-Knighton Health System followed this same course with its approach to marketing communications. This philosophy ultimately resulted in the institution becoming an early adopter of billboard advertising in the health services industry, kicking off a decades-long relationship with this particular communications medium that continues to this day [19, 22].

Willis-Knighton Health System’s use of billboard advertising began in the late 1970s when it was known as Willis-Knighton Memorial Hospital. At the time, health services establishments throughout the state of Louisiana were not using billboards to advertise their various offerings. In fact, advertising for health services, regardless of medium, was generally rare during this particular period. Healthcare institutions instead tended to rely on word-of-mouth referrals from patients and the reputations of physicians for building patient volume, with additional support being provided when newspaper, television, radio, and other media outlets would carry stories of public interest, profiling things such as innovative medical technologies newly introduced in the market, enhanced capacity afforded by a newly constructed hospital wing, and similar newsworthy items [19]. As such, the bulk of the conveyance efforts supplied by healthcare institutions fell under the public relations component of the traditional marketing communications mix, essentially entailing the packaging and distribution of stories of public interest to media outlets in hopes that the associated accounts would be presented to audiences, thus promoting given establishments [2, 9].

While Willis-Knighton had acquired a loyal following of satisfied patients and a dedicated team of skilled physicians, the growth desired by executives required the institution to reach beyond circles of existing publics and tap into new pools of prospects, calling for enhanced outreach and, naturally, an expanded media presence. As a smaller, less prominent healthcare entity, garnering media interest and attention proved to be challenging. Media outlets tended to focus their efforts on the market leader of the day, giving that particular healthcare institution the bulk of available airtime and space, reducing Willis-Knighton’s ability to be featured in associated news stories. Numerous attempts to achieve a sufficient media presence through public relations efforts yielded little, prompting executives to turn to advertising to promote the institution, with one of the most prominent and, especially in the given era, unusual selections being billboard advertising [19].

**Fig. 1:**
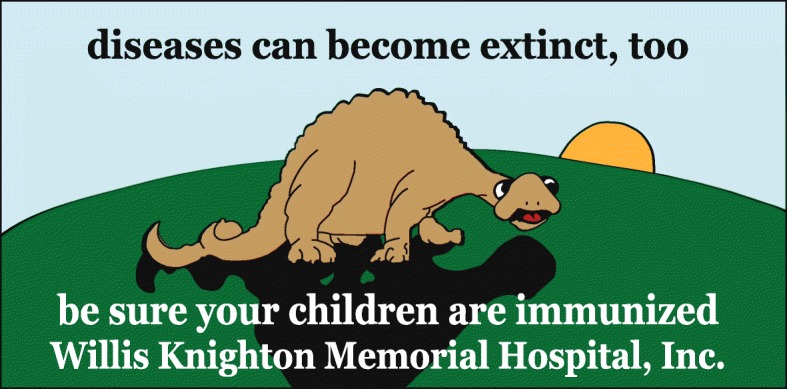
A rendering of Willis-Knighton Health System’s first billboard advertisement (ca. 1978)

For quite some time, Willis-Knighton’s executives had been observing the widespread use of billboards by retail entities, such as automobile dealers, restaurants, and gasoline stations, noting also that billboard use by many of these businesses appeared to be ongoing, indicating that advertising clients viewed the medium to deliver enough value to justify lease renewals. Since these entities were reliant on customer traffic, just as are health services organizations, executives postulated that billboards might work equally well for attracting those with medical wants and needs to healthcare facilities. As such, Willis-Knighton decided to experiment with the medium, launching in 1978 a campaign featuring the billboard advertisement presented in Fig. 1 and becoming the first hospital in the state of Louisiana to deploy billboard advertisements [19, 22].

As seen in the associated figure, the billboard advertisement featured a cartoon dinosaur and conveyed the importance of childhood immunizations, noting that diseases, too, can become extinct. The simplicity of the design, which did not even feature the institution’s logo, is indicative of an inaugural effort in a much simpler time period, but the trial proved to be successful. While the performance of this particular campaign was not formally studied, anecdotal evidence suggested that the institution’s billboards were noticed by many in the marketplace, including patients, employees, and other publics, prompting executives to continue using the given medium of communication [19].

Over subsequent decades, Willis-Knighton Health System’s billboard advertising use and experience grew. As the institution’s footprint advanced beyond its original base of west Shreveport, reliance on billboards increased, with the medium playing an integral role in conveying the story of Willis-Knighton Health System and its offerings to passersby circulating in and around newly-entered communities. Executives were pleased with the performance of the institution’s billboard advertisements, with both informal and formal studies (e.g., [15]) indicating that the medium generated an acceptable return on investment. Today, Willis-Knighton Health System leases 28 billboards in the greater Shreveport marketplace, more than any other healthcare provider in the region, with the collection containing both static and digital examples. Excellent deployment experiences have made billboards a staple of Willis-Knighton Health System’s marketing communications mix [19, 22].

## Willis-Knighton Health System as an owner-operator of digital billboards

Periodically across Willis-Knighton Health System’s many experiences using billboard advertising, executives contemplated placing billboards onsite at several of its campuses, with these thoughts being compelled by the excellent roadside visibility and high traffic counts characterizing its locations in the context of the medium’s many desirable attributes which capitalize on superior lines of sight and burgeoning traffic volume. A perfect fit seemed to exist. With on-premise signage already being robust, executives believed that by placing billboards onsite, roadside communications could extend beyond the conveyance of brand identity to supply detailed information to passersby regarding Willis-Knighton Health System’s many services, yielding mutual benefits. These musings, however, remained confined to the drawing board for many years, hampered by the billboard advertising technologies of the day which did not permit quick and convenient message alterations.

Throughout much of Willis-Knighton Health System’s history of using billboard advertising, the medium was static, with advertisements being printed on paper or vinyl after which they were affixed onsite to given display panels. With static panels, in order for message alterations to occur, new advertisements generally must be printed to replace prior ones, making for an expensive and labor intensive process. This, of course, limited their messaging potential, which in turn limited the potential derived from institutional ownership and operation. In the early 2000s, however, spurred by the declining costs of digital panels, the billboard advertising medium experienced a monumental transformation, entering the electronic age with the introduction of digital billboards. Unlike static panels which feature a single advertisement at a particular site for a given period of time (e.g., 30 days, six months), digital billboards rotate multiple advertisements at single sites, with each advertisement being featured for several seconds before cycling to the next [12, 13]. The digital format permits billboard advertisements to be altered on demand, requiring nothing more than forwarding new artwork to the given outdoor advertising company along with a request to make the alteration, affording perhaps the medium’s most notable advancement derived from its digital transformation. In the Shreveport-Bossier City market, digital billboards made their first appearance in 2006, with Willis-Knighton Health System’s first use of this platform beginning in 2008.

**Fig. 2:**
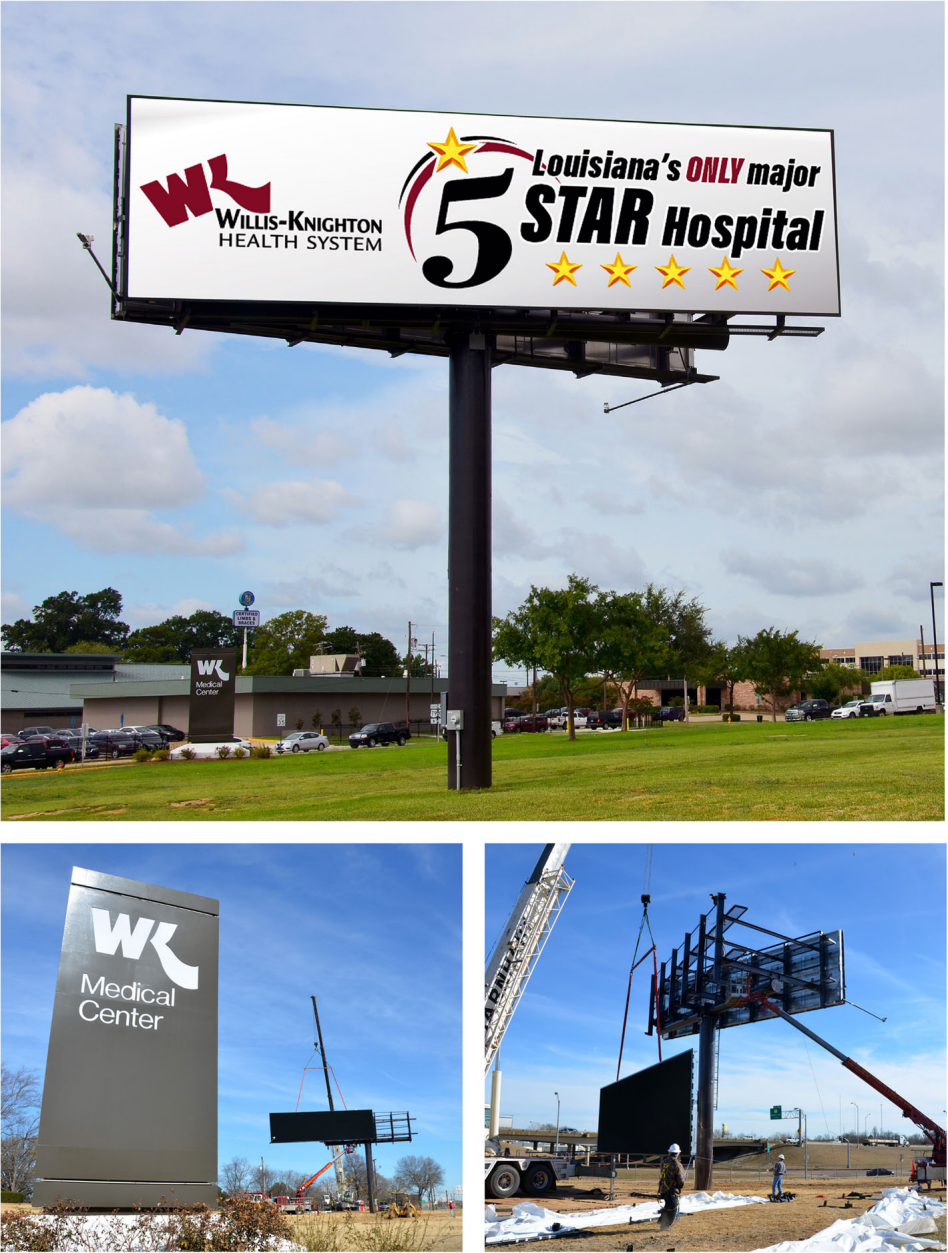
Willis-Knighton Health System’s digital billboard located on I-20 at Greenwood Road in Shreveport, Louisiana

**Fig. 3:**
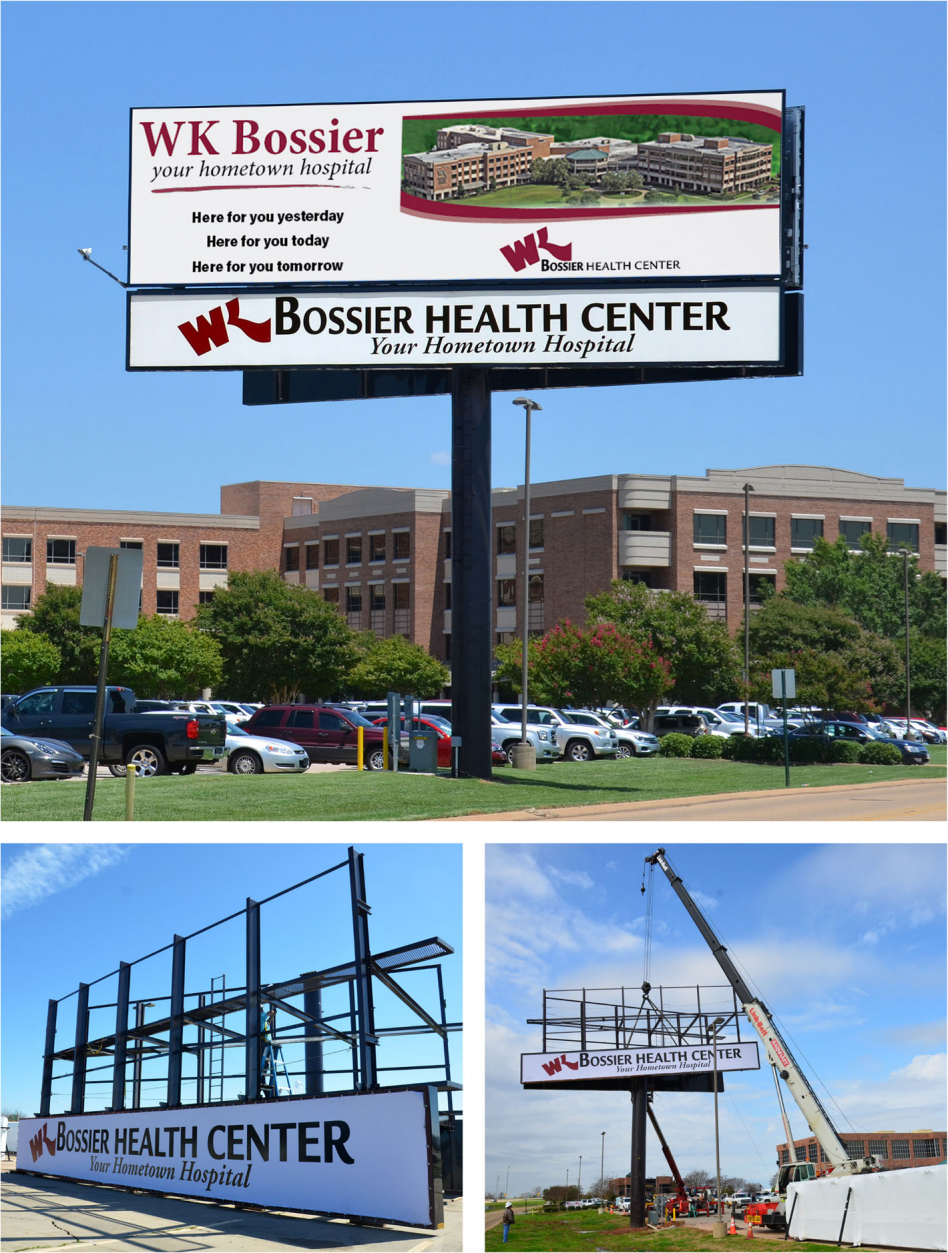
Willis-Knighton Health System’s digital billboard located on I-220 near Airline Drive in Bossier City, Louisiana

As the institution’s experience with digital billboards grew, it became apparent to executives that the advancements associated with the electronic format resolved many of the concerns which had earlier hampered pursuit of Willis-Knighton Health System’s long-running idea of installing and operating billboards on its campuses. In 2017, executives decided that the time was right for the institution to move the idea from concept to reality, and in 2018, the system installed digital billboards at several of its locations. The first one, presented in Fig. 2, is located at Willis-Knighton Health System’s main campus, Willis-Knighton Medical Center, on Greenwood Road in Shreveport. It features two 14′ × 48′ digital panels, situated on either side of the structure, capturing the attention of passersby traveling east and west on Interstate 20, as well as along area surface streets. The panels are affixed to a vee structure which angles them to enhance visibility from the roadway. A second one, presented in Fig. 3, is located at Willis-Knighton Health System’s Bossier City campus, WK Bossier Health Center, on Hospital Drive. This 2-sided billboard, structured as a vee, captures the attention of passersby traveling east and west on Interstate 220 and circulating on adjacent surface streets. A 6′ × 48′ static signage area is situated beneath each of its 14′ × 48′ digital panels, something required by Bossier City’s signage regulations. At this campus, on property fronting Airline Drive, a third digital billboard is currently under construction; a 2-sided, vee structure, featuring 12′ × 24′ digital panels, complemented by 5′ × 24′ static signage areas. Each of these billboards is controlled remotely via computer by staff members of Willis-Knighton Health System’s Department of Marketing and Public Relations which is based on its main campus. Willis-Knighton Health System has been most impressed with its new advertising platforms, so much so that other digital billboards are planned for construction in coming months.

## Opportunities

Healthcare institution-owned and operated digital billboards permit communications power previously only held by media firms, providing enormous communications utility, courtesy of the medium’s ability to influence passersby on an on-demand and continuous basis. In its brief experience as an owner-operator of digital billboards, Willis-Knighton Health System has noted that the medium supplies a range of opportunities, as follows.

### Conveyance of messages continuously, around-the-clock

In cases where digital billboards are leased from outdoor advertising companies, since multiple slots are featured at each digital billboard location, advertisers desiring 24–7, uninterrupted message displays must lease all available advertising slots. This very often is not possible due to existing leases, and even if all slots are available, the cost almost certainly will be catastrophically high. In the greater Shreveport marketplace, for example, a single advertising slot on a digital billboard situated along a high-traffic corridor can cost around $3000 per month, with panels usually rotating 6–8 slots. Digital billboard ownership removes the competition for slots, as given platforms are fully controlled by the owner-operator, permitting the circulation of messages continuously, around-the-clock. This benefit also permits healthcare institution-owned billboards to play significant roles in reinforcing the effects of other advertising media in use at given times, bolstering the aggregate strength of marketing communications efforts. Further, in cases where competing healthcare organizations do not possess an equivalent asset, a significant competitive advantage is afforded, thanks to the exceptional communications potential provided by owned digital billboards.

### Message alterations on-demand, instantaneously

The ability to change advertising messages remotely whenever desired represents a key advantage of digital billboards, whether leased from outdoor advertising companies or institutionally owned. However, when digital billboards are institutionally owned, message alterations can be instantaneous, as there is no need to route the new advertisements to an external entity (i.e., an outdoor advertising company) for uploading. While outdoor advertising companies address change requests quickly, often within 24 h of submission, a completely internal operation affords immediate alterations. At Willis-Knighton Health System, the placement of new advertisements on its digital billboards literally takes seconds, permitting the instant dissemination of messages to passersby.

This ultra-fast, remote, message alteration capability is certainly beneficial for the conveyance of marketing communications, but it also can be helpful in situations far beyond matters of institutional promotion. Consider emergent situations experienced in a given community (e.g., natural disasters, terrorist attacks), requiring evacuations of the populace which result in roadways being overrun with vehicles. In such cases, the institution can forgo placement of promotional messages on its digital billboards, replacing these with critical public service announcements, providing passersby with helpful and potentially life-saving information. Since many healthcare institutions operate around-the-clock and remain operational even in the worst of situations, messages can be presented and updated continuously throughout the given calamity experienced. Willis-Knighton Health System has yet to use its digital billboards in this fashion, but it is prepared to do so whenever warranted. This is one of many ways that healthcare institution-owned digital billboards can be used in unique and helpful manners well beyond the realm of traditional marketing communications.

### Presentation of messages in immediate proximity to points of service

Unlike leased digital billboards which are situated offsite, often at least some distance from the advertising entities providing the promoted services, onsite digital billboards present messages directly at the point of service. This point-of-service display aids consumers in linking healthcare establishments with services offered. It also fosters opportunities for immediate exchange, as in cases where individuals transiting by a given digital billboard have a medical need (e.g., a flu shot), see a message conveyed via billboard which can fulfill that particular need (e.g., “Get your flu shot today at WK Bossier Health Center!”), and decide to stop by for the noted service. Messages such as ER and medical clinic wait times assume new value when being conveyed at the location of service, with these details also serving to facilitate exchange. Further, this proximity attribute, when combined with other features of digital billboards, can positively influence other initiatives. Nurse recruitment advertisements, for example, can be timed to be displayed during shift changes occurring at facilities across the community, providing opportunities to attract the attention of caregivers commuting to and from competing institutions, letting them know that opportunities are available at that particular time and place.

### Reduced dependency on media firms for messaging

By installing and operating digital billboards, healthcare establishments are less reliant on media firms for the delivery of advertising messages. This reduced dependency translates into fewer media buys, diminishing the magnitude of advertising expenditures. Cost savings here, of course, are offset by the costs of acquiring and operating digital billboards, but such expenditures must be considered in the context of the benefits afforded by possessing full command and control of a commercial advertising platform. In Willis-Knighton Health System’s case, digital billboards were acquired primarily to complement, rather than reduce or eliminate, existing advertising, but the ability to reduce media buys and associated advertising expenditures remains a useful option afforded by digital billboard ownership. Perhaps more compelling from Willis-Knighton Health System’s perspective is that reduced dependency on media firms lessens concerns regarding potential digital billboard inventory shortages. Especially in competitive markets, it is not uncommon for digital billboard availabilities in desirable areas to be limited or nonexistent at given times, hampering the marketing communications plans of institutions shut out of advertising at preferred locations, further bolstering the benefits afforded by digital billboard ownership.

## Obstacles

While Willis-Knighton Health System’s experience as an owner-operator of digital billboards has demonstrated a number of opportunities, obstacles indeed exist and must be factored into consideration by any entity seeking to establish an institution-owned, roadside advertising presence. Fortunately, Willis-Knighton Health System was able to circumvent obstacles to realize digital billboard ownership, but associated barriers can be quite daunting and possibly even impossible to overcome, with each case being situation dependent. Potential obstacles include the following.

### Cost factors associated with acquisition and operation

A digital billboard which notably includes a steel monopole, posting structure, and massive digital panel understandably is very expensive. Pricing, of course, depends on specifications desired (e.g., panel size, quantity, resolution; monopole height, cladding; structure design, platform characteristics; etc.), but Willis-Knighton Health System’s recent experience suggests that a nicely-optioned, 14′ × 48′, single-sided digital billboard from a reputable manufacturer generally can be acquired and installed for around $250,000. Further, establishments must also consider operating requirements, such as electricity costs for powering the digital billboard, cellular connection costs for controlling the platform remotely via computer, routine maintenance costs for the structure itself and its digital panel, panel replacement costs at the expiration of its lifespan, and personnel costs associated with acquiring and retaining staff members responsible for operating the digital billboard. Such costs certainly can prohibit many healthcare institutions from pursuing digital billboard ownership and operation, but for those that possess the requisite funds and anticipate benefiting from the associated attributes, digital billboards can serve as useful communications investments.

### Zoning barriers which limit or prevent installations

Municipalities generally have very defined guidelines governing all aspects of signage in their given jurisdictions, with designated bodies (e.g., metropolitan planning commissions) being responsible for reviewing signage applications and issuing permits to successful applicants. As sign regulations emerge from political processes, variation between and among communities should be expected, with some municipalities being more restrictive than others. In general, however, guidelines will address things such as permissible sizes and spatial characteristics (e.g., roadside setback, elevation, and spacing requirements). Since signage policies are municipality-dependent, only by investigating regulations in the particular community in which installation is desired can one ascertain the viability of a given application. If digital billboards are not allowed by current regulations at selected sites, possibilities still might exist for their installation by applying for a variance which essentially is an appeal requesting special permission to install a nonconforming sign. Regardless, there are no guarantees that application reviews, including any associated appeals, will conclude with a permit being issued, creating perhaps the most pressing obstacle faced by healthcare entities desirous of installing and operating digital billboards.

### Views held by the public which are not supportive of billboards

It should be noted that billboard advertising is not without its controversies, with these tending to circulate around perspectives that the medium is harmful to scenic beauty (e.g., by blocking natural roadside terrain), the environment (e.g., by removing trees to permit installations), and motorist safety (e.g., by creating driver distractions) [23, 24]. These views, which generally have been refuted by the outdoor advertising industry and others [25–27], do not appear to be particularly widespread or effective, evidenced by the continued proliferation of billboards. Such perspectives, however, illustrate that at least some view the medium to be undesirable which, in turn, could negatively influence their perceptions of healthcare entities which own and operate digital billboards. Fortunately, Willis-Knighton Health System exists in a billboard-friendly marketplace, generally free of signs indicating animosity toward the medium (e.g., vandalism, protests). Further, in an earlier study examining the effectiveness of one of its billboard advertising campaigns, Willis-Knighton Health System discovered that its patients possessed favorable views of the medium, giving further assurances that the institution’s use of billboards is noncontroversial [15]. Still, healthcare entities would be wise to examine their given communities to ensure that they are billboard-friendly before pursuing ownership.

## A protocol for pursuing digital billboard ownership and operation

**Fig. 4:**
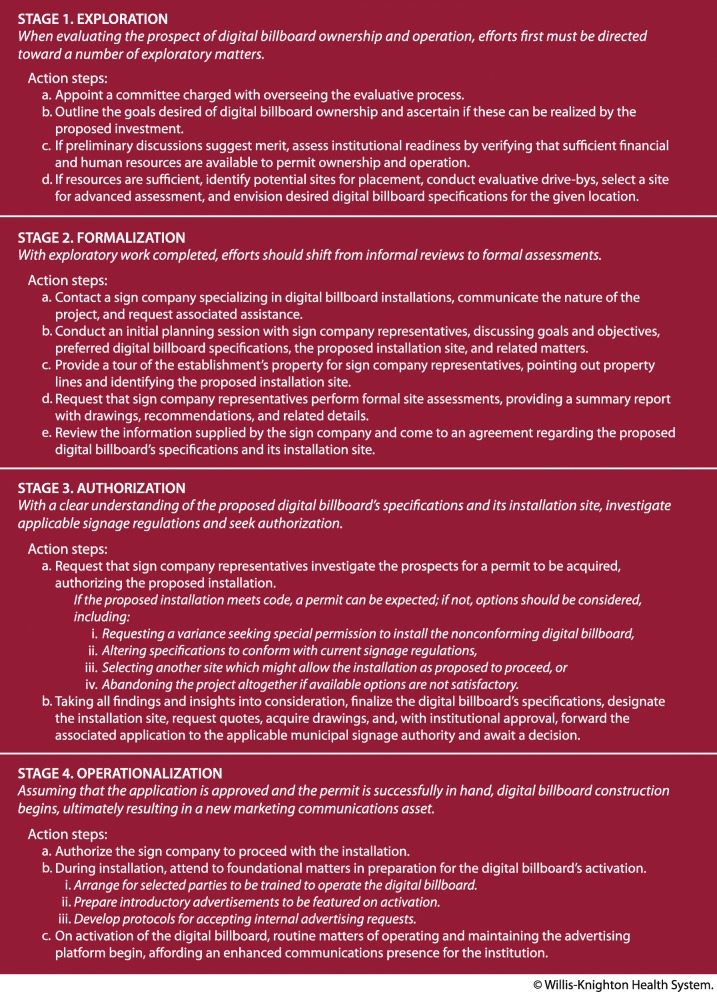
A protocol for pursuing digital billboard ownership and operation

Without any prior experience owning and operating digital billboards, Willis-Knighton Health System’s executives needed to craft an associated protocol for pursuing the concept. After significant discussion and debate, a framework emerged which guided the institution through various considerations and assessments leading to the installation of its digital billboards. Presented in Fig. 4, this plan can be used by most any healthcare institution seeking to determine the merit of entering the world of outdoor advertising as an owner-operator of digital billboards. The stages of this protocol are as follows.

### Stage 1: exploration

When evaluating the prospect of digital billboard ownership and operation, efforts first must be directed toward a number of exploratory tasks. The first step involves appointing a committee charged with overseeing the process. Committee composition is dependent on the desires of the given establishment, but it obviously must include representation from those responsible for marketing, as their insights on advertising will be critical and they likely will be responsible for operating the proposed digital billboard. Since digital billboard construction is intensive, involving assessments ranging from soil tests to ensure that the structure can be supported to electrical assessments to ensure that the panels can be powered, it is advised that engineering and construction personnel be included on the committee. Further, due to the high costs associated with digital billboard ownership and operation, it is advised that senior leaders, most notably the healthcare establishment’s top officer, serve on the committee. Additional perspectives from a range of occupational categories (e.g., administrators, physicians, nurses, technicians) also can be helpful, especially for envisioning the impact of the digital billboard on operations and ascertaining the ideal location for its placement.

Once the committee’s composition is formulated, the group should convene for its initial meeting, with its first matter of business being exploration of the concept of digital billboard ownership and operation in full, seeking specifically to determine if the pursuit is expected to be worthwhile. Here, the committee must outline the goals desired of digital billboard ownership and ascertain if these indeed can be realized by the investment. The institution’s prior history of using billboards, if any, should be discussed, with candid assessments offered regarding associated promotional experiences and outcomes. If these preliminary discussions suggest merit, attention then should be directed toward assessing the institution’s readiness for this endeavor by verifying the sufficiency of resources to permit the digital billboard’s purchase and operation. Extensive financial resources obviously are required for such pursuits. Early in the evaluative process, without digital billboard specifications in hand, only rough estimates of costs will be possible, but these can be acquired easily by contacting a specialty sign company and making a general inquiry, giving the committee at least a rough idea of what to expect. Institutions also need to ensure that they have employees on staff who possess the time and talent required to create digital artwork and post it in accordance with designated protocols. Existing marketing departments within healthcare institutions likely will already possess the talent, but capacity also must be investigated to ensure active management of the proposed digital billboard.

Assuming the presence of sufficient resources, efforts of the committee are then directed toward determining a potential location for the installation of the proposed digital billboard. For this activity, a campus map which clearly shows property lines is most helpful, as it will permit committee members to observe formal boundaries in the context of area roadways and the institution’s buildings to form a short list of prospective locations. Committee members then should engage in actual drive-bys of candidate sites, taking steps to identify the ideal selection for the proposed installation. The site selected for advanced assessment ideally should be visible from a range of distances, permitting greater opportunities for passersby to be exposed to advertising messages, and it should be expected to remain obstruction-free over time (i.e., free of potential future encumbrances caused by things such as vegetation growth, new construction, and so on which might block the proposed digital billboard). Lastly, taking the prospective site into consideration, committee members must envision desired specifications (e.g., size and number of digital panels, structure design, elevation, etc.) for the proposed digital billboard.

### Stage 2: formalization

With exploratory work completed, efforts should shift from informal reviews to formal assessments. This is accomplished most easily by contacting a sign company specializing in digital billboard installations, communicating the nature of the project, and requesting associated assistance. An initial planning session permitting the committee to meet with sign company representatives to discuss goals and objectives, preferred digital billboard specifications, the preliminary site selection, and related matters will quickly advance the given project. Importantly, committee members should accompany sign company representatives on a tour of the establishment’s property, pointing out property lines and identifying the proposed installation site. Sign company representatives will conduct further assessments independently, typically photographing the location from multiple street-level distances, analyzing lines of sight to ensure that the proposed location for the digital billboard will deliver maximum visibility from the roadway, preparing sample depictions illustrating how the digital billboard will look if installed at the particular site, and suggesting any locations observed which offer improved lines of sight over the initial selection. The committee’s final task during this stage is to review the information supplied by the sign company and come to an agreement regarding the proposed digital billboard’s specifications and its installation site.

### Stage 3: authorization

With a clear understanding of the proposed digital billboard’s specifications and its installation site, the sign company then will investigate applicable signage regulations to determine the prospects for installation. If the project meets existing codes, an associated permit from the applicable municipal signage authority can be expected. However, if the project does not conform with prevailing regulations, a permit should not be expected, warranting the exploration of options. Applicants might, for example, request a variance seeking special permission to install the nonconforming digital billboard, alter the signage specifications to conform with current guidelines, select another site which might allow the installation as proposed to proceed, or abandon the project altogether if available options are not satisfactory. Here, an experienced sign company will prove to be an invaluable resource in navigating through the associated legal framework. Taking all findings of the sign company into consideration, together with the desires of the healthcare establishment, the committee must finalize the digital billboard’s specifications, designate the installation site, request quotes, acquire drawings, and, with institutional approval, forward the associated application to the applicable municipal signage authority and await a decision.

### Stage 4: operationalization

Assuming that the healthcare establishment’s application for installation of the proposed digital billboard is approved and the permit is successfully in hand, billboard construction can proceed; a process that, weather permitting, usually takes approximately eight weeks. Matters at this point generally are left to the sign company, working in tandem with the establishment’s engineering and construction professionals who will arrange for the delivery of power to the site and address any matters associated with the installation that go beyond the authority of the sign company. While awaiting completion of the installation, staff members responsible for operating the digital billboard should be trained to use the associated software which controls panel presentation attributes. Attention also should be directed toward preparing a series of introductory advertisements which will be featured as soon as the digital billboard becomes operational. If desired, protocols for accepting internal requests for particular advertisements to be featured on the digital billboard can be developed in advance of activation, ensuring an orderly process as the institution integrates its newfound communications resource into its marketing operations. Once foundational matters are addressed and the digital billboard is activated, routine matters of operating and maintaining the advertising platform begin, affording an enhanced communications presence for its new owner-operator.

## Conclusions

Digital billboards offer significant advancements over their static counterparts and, when paired with excellent sites located on the premises of healthcare institutions, myriad communications possibilities result. By installing and operating digital billboards onsite, health and medical establishments gain extraordinary communications power and utility, permitting the active engagement of passersby in immediate proximity to their given facilities. Courtesy of their robust size and vibrant displays, these modern outdoor advertising platforms garner significant attention, something enhanced further by the ease of modifying their advertising messages, permitting new content to be delivered instantaneously. Location and resource requirements certainly will prohibit some facilities from pursuing such, but for those healthcare institutions which have suitable sites and the associated means, digital billboards offer an avenue of extreme communications opportunity.

Long-time user and proponent of billboard advertising, Willis-Knighton Health System, in seeking to amplify its promotional efforts, arrived at the notion of taking advantage of the excellent roadside visibility and high traffic counts characterizing its campuses by installing this relatively new outdoor advertising technology at several locations, effectively becoming an outdoor advertising company of sorts. The institution’s experience as an owner-operator of digital billboards has been highly fulfilling, prompting executives to plan additional installations in the near future which will further bolster the institution’s ability to engage audiences. Through the guidance supplied in this article, healthcare entities desirous of following a similar course have at their disposal a detailed roadmap for doing so, along with information regarding associated opportunities and potential obstacles. Given the importance of communicating with current and prospective patients, especially in light of the competitive nature of the healthcare industry, health and medical establishments would do well to incorporate, when and where possible, innovative communications options. Digital billboards represent one such option worthy of consideration.
